# Prognosis of hepatocellular carcinoma patients with bile duct tumor thrombus after hepatic resection or liver transplantation in Asian populations: A meta-analysis

**DOI:** 10.1371/journal.pone.0176827

**Published:** 2017-05-04

**Authors:** Chenglin Wang, Yu Yang, Donglin Sun, Yong Jiang

**Affiliations:** Department of Hepatobiliary Surgery, The Third Affiliated Hospital of Soochow University, Changzhou, P. R. China; Virginia Commonwealth University, UNITED STATES

## Abstract

**Background:**

Hepatocellular carcinoma (HCC) with bile duct tumor thrombus (BDTT) in the clinic is rare, and surgical treatment is currently considered the most effective treatment. However, the influence of BDTT on the prognosis of HCC patients who underwent surgery remains controversial in previous studies. Therefore, this paper uses meta-analysis method to elucidate this controversy.

**Methods:**

In this study, we conducted a literature search on databases PubMed, Embase and Web of Science from inception until September 2016. Each study was evaluated with Newcastle-Ottawa Scale (NOS). The pooled effect was calculated, and the association between BDTT and overall survival (OS) or disease-free survival (DFS) was reevaluated using meta-analysis for hazard ratio (HR) and 95% confidence interval (CI).

**Results:**

A total of 11 studies was included containing 5295 patients. The (HR) for OS and DFS was 3.21 and 1.81, 95%CI was 2.34–4.39 and 1.17–2.78 respectively.

**Conclusions:**

The results showed that HCC patients with BDTT had a worse prognosis than those without BDTT after hepatic resection or liver transplantation (LT).

## Introduction

Hepatocellular carcinoma (HCC) is a solid malignant tumor often caused by hepatitis virus infections, cirrhosis, alcohol abuse, and obesity. It is a major health problem and the third most common cause of cancer-related death[1]. The incidence of HCC is increasing worldwide, especially in Asia-Pacific countries, of which China alone contributes half of the total number of cases[2]. According to statistics, about 600,000–700,000 people yearly died for HCC in the world[3]. Although the diagnosis, treatment and surgical techniques have been improved greatly in the past few decades, the prognosis of HCC is still poor with 5-year survival rate around 5~6%[4]. Generally, HCC spreads through the liver via the portal vein. Thus, portal vein tumor thrombus is commonly observed in radiological images and resected liver samples[5]. However, HCC with bile duct tumor thrombus (BDTT) is rarely seen in clinical practice and accounts for only 1.2–12.9% of total HCC cases [6]. When BDTT occurs in major bile ducts, jaundice is the main clinical manifestation and can be easily misdiagnosed as a bile duct cancer or biliary stones. At present, the diagnosis of BDTT mainly depends on clinical symptoms, physical examinations, image studies, surgical exploration and postoperative pathological results. Although BDTT is known as a risk factor of the prognosis of HCC patients, the underlying mechanisms are still not clear [7]. The palliative treatments, such as transcatheter arterial chemoembolization (TACE), internal biliary stenting and radiotherapy, often lead to disappointing outcomes. The efficacy of hepatic resection or liver transplantation (LT) for long-term survival of HCC patients with BDTT is controversial[8]. Therefore, we performed a meta-analysis to evaluate the prognosis of HCC patients with BDTT after hepatic resection or LT.

## Materials and methods

### Literature search strategy

A protocol before performing statistics was developed. In detail, systematic search was performed for Pubmed, Embase and Web of Science by two researchers and the relevant studies published from inception until January 2016 were gathered by the following terms: (BDTT OR BDT OR bile duct tumor thrombus) AND (prognosis OR survival OR mortality OR prognostic) in combination with (HCC OR hepatocellular carcinoma OR liver cancer OR hepatoma). In addition, all references in the relevant studies were also manually examined. The citations were managed using Endnote X7 software.

Eligible studies met the following criteria: 1) pathological diagnosis of HCC was confirmed by immunohistochemistry after hepatic resection or liver transplantation (LT), and BDTT was found during surgery or in postoperative histological analysis; 2) studies analyzed the relationship between HCC with BDTT and the survival of HCC patients; 3) studies provided hazard ratio (HR) for overall survival (OS) or disease free survival (DFS) and 95% confidence interval (CI) or included effective information to calculate HR and 95% CI; 4) sample size was at least 20. In the case of studies with overlapping populations, the largest number of cases was included for meta-analysis.

Studies with focus on non-primary liver cancer published in languages other than English and unable to extract enough information to calculate HR or 95% CI were excluded.

### Data extraction and qualitative assessment

Retrieved studies were reviewed by two independent researchers to extract raw data according to a standard data-collection protocol including first author name, publication year, regions, patient characteristics (number, gender and age), Child-Pugh grade, follow-up data, method of therapy, HR value and 95% CI. Their discrepancies were discussed and resolved seriously. The quality of studies was independently assessed by two researchers using the Newcastle-Ottawa Quality Assessment Scale (NOS) with a maximum score of 9.

### Statistical analysis

Data analyses were performed using Stata 12.0 software. Data were pooled to analyze their prognostic value to predict the OS of HCC patients with BDTT. The HR and 95% CI were calculated from Kaplan–Meier survival curve using Engauge Digitizer version 4.1 software. Q and I^2^ values were used to assess the degree of statistical heterogeneity using a random effect model if P < 0.1 or I^2^ > 50%, otherwise using a fixed-effect model. In addition, Begg’s funnel plot test was used to examine publication bias for the studies included.

## Results

### Study selection and characteristics

**Fig 1:**
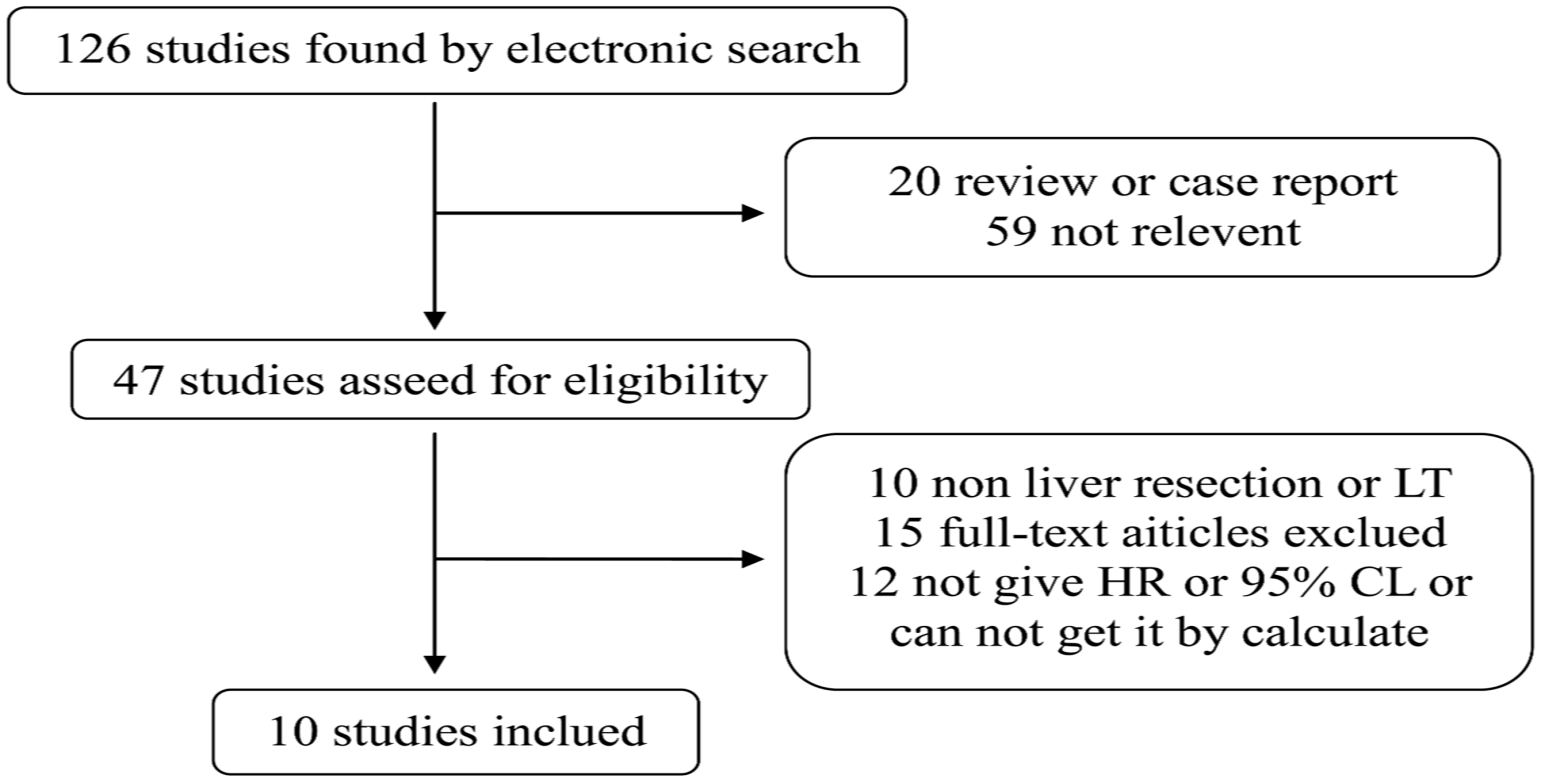
Flow diagram of the study selection process.

Fig 1 shows the search and filtering process. 126 studies that met the inclusion criteria and published from 2000 to 2015 in English were found by electronic search using the above-mentioned keywords. Of them, 10 studies of 4869 patients that met the requirements for meta-analysis were further analyzed in detail[1, 3, 7–14]. Table 1 and S1 Table lists the main features of the 10 studies included including study location, average age of patients, followup period, etc. It is clear from the table that 1) all studies were performed in Asia; 2) the median follow up period was reported in only 6 studies and the followup period was from 1 to 156 months in one study. The quality scores was ranged from 4 to 8. All patients had undergone a hepatic resection and LV was performed in one patient.

**Table 1:** Baseline characteristics for studies included in meta-analysis.

Rsfs.	Year	Area	Gender (W/M)	Age (Year)	HBV(+) (%)	Child-Pugh Grade (A/B/C)	Follow-Up (Month)	Therapy	Survival Analysis
Yu et al[12]	2010	China	582/94	50	NR	600/NR/NR	1–156	Resection/LT	OS
Satoh et al[9]	2000	Japan	535/136	60.6	18.5	NR	NA	Resection	OS
Shiomi et al[10]	2001	Japan	111/21	59.8	20	NR	NA	Resection	OS
Yeh et al[11]	2004	Taiwan	NR	NR	NR	NR	25.1	Resection	OS/DFS
Noda et al[7]	2011	Japan	450/101	58	48.8	458/93/0	35	Resection	OS
Shao et al[8]	2011	China	256/41	51	94.3	NR	39	Resection	OS
Oba et al[3]	2014	Japan	663/133	64.9	19.6	702/94/0	47	Resection	OS
Kim et al[13]	2014	Korea	63/30	55	81.7	NR	NA	Resection	OS/DFS
Wong et al[14]	2014	China	208/51	56.1	80	240/23/0	24.9	Resection	OS/DFS
Pang et al[1]	2015	China	354/97	51.7	92.8	802/149/0	96	Resection	OS

NR: not reported, NA: not applicable

### Pooled HR value for OS and DFS

**Fig 2:**
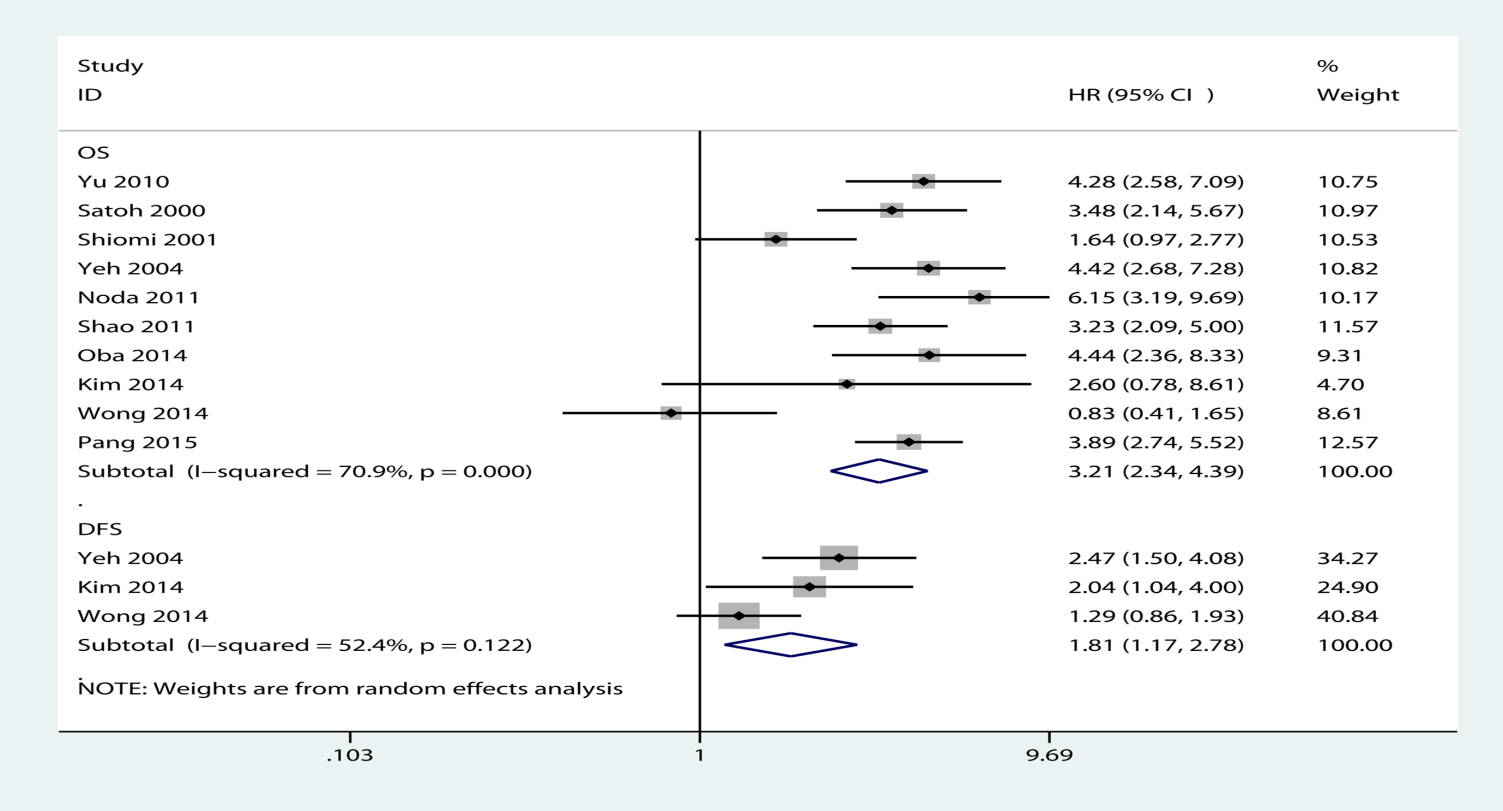
Forest plots of studies evaluating hazard ratios of hepatocellular carcinoma (HCC) with bile duct tumor thrombus (BDTT) after hepatic resection or liver transplantation (LT) for overall survival or disease-free survival.

In the meta-analysis, the heterogeneity among the included studies was significant (I^2^ > 50%). Thus, the random-effect model was employed to evaluate the summary. The results showed that HCC patients with BDTT had a poor prognosis after hepatic resection or LT. The pooled HR for OS and DFS was 3.21 (95% CI, 2.34-4.39) and 1.81 (95% CI, 1.17-2.78), respectively. The degree of between-study heterogeneity was observed for OS (I^2^ = 70.9%, P<0.001) and DFS (I^2^ = 52.4%, P = 0.122) (Fig 2).

### Analysis of heterogeneity, publication bias and sensitivity

**Fig 3:**
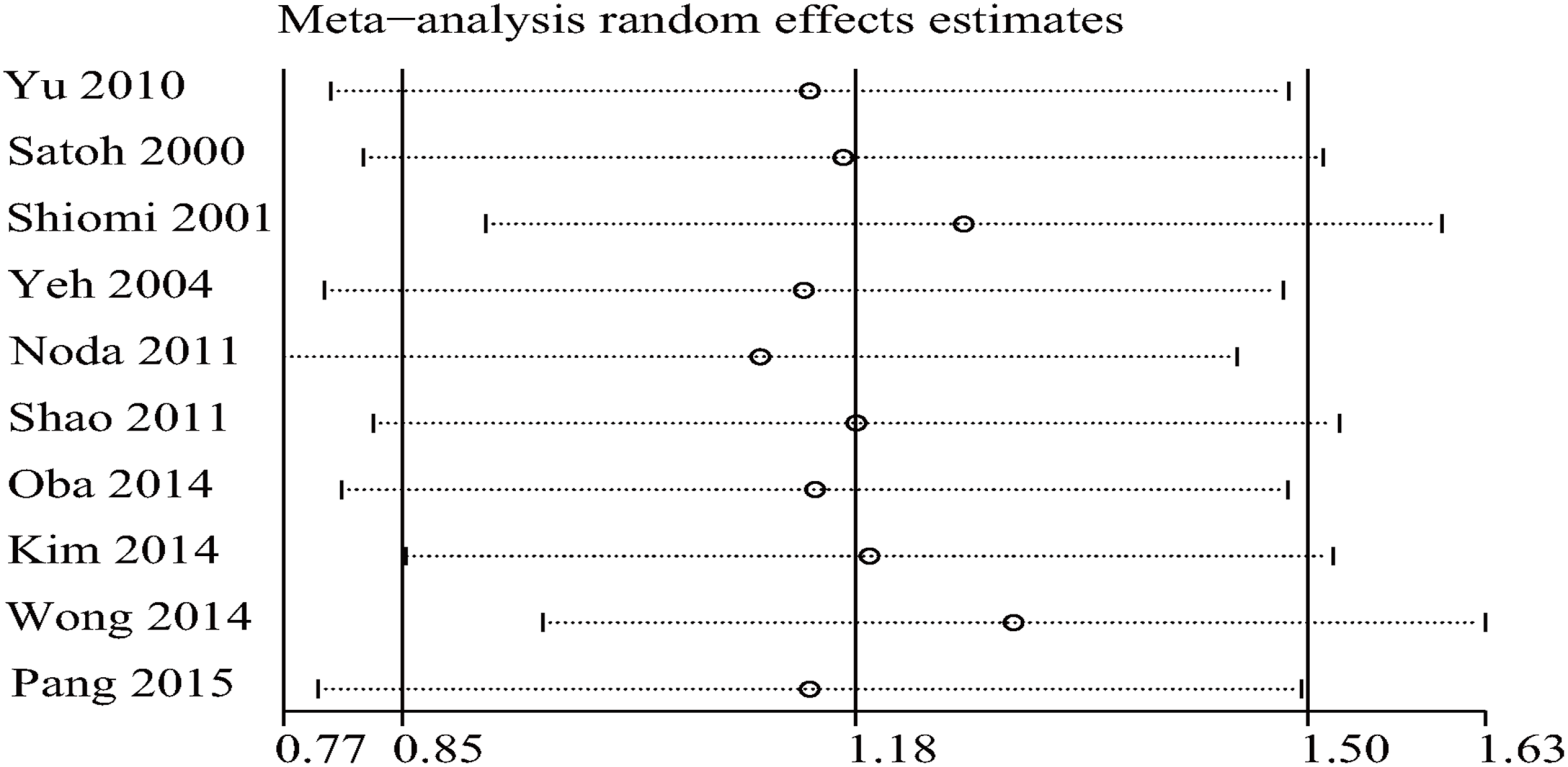
Sensitivity analysis for the evaluation of heterogeneity.

**Fig 4:**
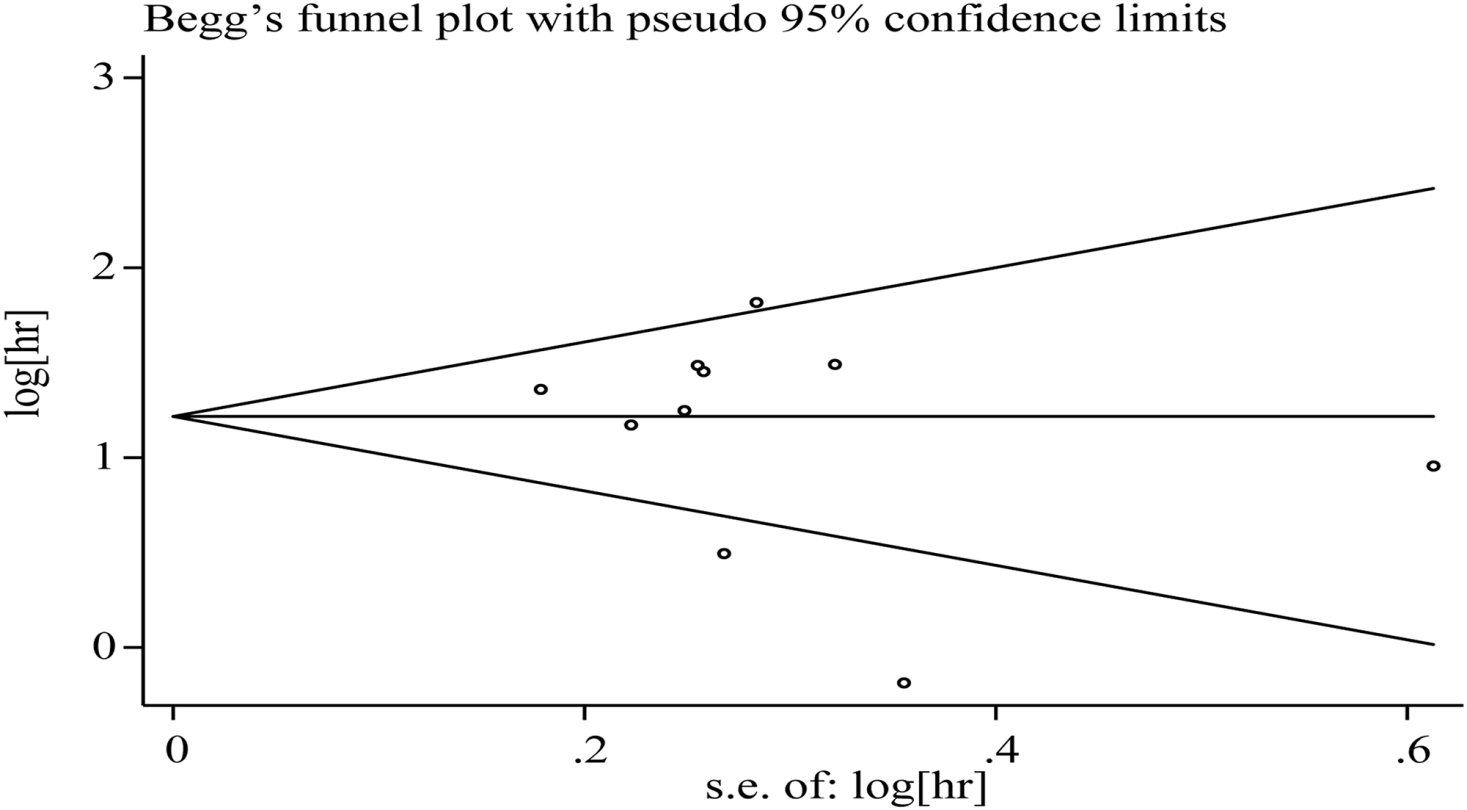
Funnel plots for the evaluation of potential publication bias.

The heterogeneity of the study may be related to 1) these studies were from different countries, 2) different characteristics of the population (for example, HBV positive rate was significantly higher in the studies from China and Korea than Japan) and 3) the technical level of the surgeons is different. Sensitivity analysis showed that two studies (Wong 2014 or Shiomi 2001) might affect the pooled assessment (Fig 3). After removing one study (Wong 2014), the heterogeneity was reduced (OS: I^2^ = 44.5%, P = 0.72), and the results were positive (HR: 3.65; 95% CL, 2.89–4.46). When the two studies were excluded, there was no heterogeneity and the pooled HR for OS became 4.01 (95% CL, 3.36–4.79). Therefore, our results are of significance. Begg’s funnel plot was conducted to detect the publication bias (Fig 4). The results showed that there was no significant evidence of publication bias (p = 0.693).

## Discussion

HCC with BDTT is an uncommon disease and the first case was reported in 1947[15]. It reported a case of obstructive jaundice caused by thrombus when HCC tumor cells invaded into the bile ducts. Lin et al. proposed the clinical classification of icteric type hepatoma in 1975. In the following years, more case of HCC with BDDT were published[9, 10, 16–18]. According to previous reports, the incidence of HCC with BDTT was relatively low[19, 20]. A study reported that the incidence of BDTT was 3.4%, while it was only 1.4% for macroscopic BDTT[3].

Previous studies have shown that BDTT can occur even when the primary tumor was at an early stage[21, 22]. However, the mechanism underlying occurrence of BDTT remains unclear. There are three possible mechanisms for HCC invading into biliary system. First, bile ducts are filled with continuously growing distal tumor cells; Second, the distal common bile duct is obstructed by floating tumor debris and blood clots; Third, the bile ducts were partially or completely obstructed by clots from hemorrhaging tumor sites[16, 23, 24]. Edmondson and Steiner described the tumor thrombus in the biliary system as grayish white, fragile, and chicken fat-like[15]. BDTT in extrahepatic bile duct was discontinuous and usually grew faster than primary tumors. Tumor thrombi rarely invaded into the sub-mucosa of large bile ducts and generally did not adhere to the bile duct wall[8, 25]. As a result, surgeons could easily remove the thrombus from bile duct.

Currently, the origin of tumor cells in BDTT is not yet clear. There are two hypotheses. One holds that tumor cells are derived from mature tissue cells; The other proposes that tumor cells are originated from stem cells. Lee et al. showed that the prognosis of HCC patients with hepatic stem cells gene expression pattern was poorer than HCC patients with mature cells gene expression pattern[26]. Liu et al. revealed that in vitro cultured human intrahepatic biliary epithelial cells have some common characters of hepatic stem cells, such as the expression of ALB, AFP, dry cell factor receptor (c-kit), cytokeratin and so on, and this findings support the hypothesis that hepatic stem cells are present in intrahepatic bile duct system[27]. It has been found that the proto-oncogene Bmi1 is highly expressed in hepatocellular carcinoma [28, 29]. Zhang et al. also disclosed that rat oval cells with high expression of Bmil can differentiate into a low degree of hepatic tumor cells in nude mice, suggesting that hepatic stem cells may transform into liver cancer stem cells. Yu et al. reported that liver stem cell markers c-kit, CD 90, CD133 and epithelial cell adhesion molecule were overexpressed in tumor thrombus in the bile ducts of primary HCC patients. In the light of the small HCC tumors and the fast growing tumor thrombus in the bile ducts, these tumors cells may originate from the intrahepatic bile duct cancer stem cells[12].

HCC with BDTT is clinically featured as liver tumor with biliary dilation associated often with hepatitis B virus infection and to some extent with cirrhosis and has increased serum AFP and ALP levels. HCC patients with BDTT often have obstructive jaundice, bile ducts infection, upper abdomen discomfort or right hypochondrium pain. Some patients may have no clinical manifestations of jaundice in the visit. With disease progressing and bilirubin level increasing, the symptoms of jaundice can also be alleviated because the tumor thrombus is partly necrotized and detached. Patients can also be diagnosed as a high fever, abdominal pain, or a typical Charcot triad which conceals the existence of liver tumor[30]. As HCC often associates with liver cirrhosis or biliary obstruction, the serum levels of ALT, AST, TB and ALP are elevated to varying degrees, and HBV serological markers are mostly positive. Moreover, AFP and CA19-9 levels are simultaneously increased in most patients. The lower pathologically differentiated HCC with BDTT is often associated with vascular invasion. Hepatoma cells are the main component of tumor thrombi and often mixed with white blood cells. Bile ducts could be blocked by cancer cells with clots when HCC cells invaded into the bile ducts and caused bleeding[31].

Many methods have been used to treat HCC patients with BDTT, including hepatoectomy, biliary tract drainage, percutaneous transhepatic cholangial drainage (PTCD), alcohol injection, hepatic arterial chemoembolization, local radiation and anti-viral therapy. Among them, the most effective one is hepatoectomy plus thrombus removal[32]. The surgical principle is radical resection of primary tumor and biliary tract thrombus and full drainage of the bile ducts[33]. However, the prognosis of HCC patients with BDTT after hepatic resection is still controversial. Yeh et al. and Yu et al. reported that HCC patients with BDTT encountered a worse prognosis than patients without BDTT[11, 12]. Other previous studies revealed no differences in prognosis between them[9, 10]. In order to address the controversy, we analyzed and summarized all the studies available through meta-analysis and concluded that HCC patients with BDTT have a worse prognosis after hepatic resection or LT (HR = 3.21, 95% CL, 2.34–4.39).

In addition, LT for treatment of HCC with BDTT has also been of great concern. The high possibility of HCC recurrence after LT was the main problem, even though HCC consisted to Milan criteria was an indicator for LT. Ikenage et al. reported that the recurrence rates and invasion nature of HCC patients with BDTT were significantly higher than patients without BDTT[18]. Therefore, most surgeons are cautious about performing liver transplantation for HCC patients with BDTT. However, as a new surgical approach, liver transplantation for HCC patients with BDTT who have lost the chance of conventional hepatectomy deserves our further study and exploration.

The recurrence of HCC is the main cause of death after hepatectomy[34, 35]. In spite of the same treatment, the recurrence of HCC patients with BDTT is even earlier[36]. 53% of HCC patients with BDTT relapsed in three months after surgery[18].

The studies included in this study are consistent with the inclusion criteria and the results are reliable. In addition, all included studies were from Asia, so the results may be more applicable to the Asian region. To obtain more comprehensive and accurate results, large-scale prospective studies are needed. Although we have comprehensively evaluated the prognosis of patients after resection, the meta-analysis has some limitations. Firstly, the study did not include symposium reports and other unpublished articles. Second, all studies included are published in English, and there may be a language bias.

## Conclusions

This analysis showed that patients with BDTT had a worse prognosis after hepatectomy or LT compared with HCC patients without BDTT. Although the prognosis of patients with HCC combined with BDTT who underwent surgical treatment is discouraging, it is better than in patients who have not undergone surgical treatment; and jaundice is not a late symptom or surgical contraindication, and a safe choice of safe surgical procedure can prolong patient survival. Surgery is still considered a first-line treatment for those patients that is tolerant to surgery.

## Supporting information

S1 Table HR, LL and UL for studies included in meta-analysis. (DOCX)
