# Supplementary material for: Prognosis of hepatocellular carcinoma patients with bile duct tumor thrombus after hepatic resection or liver transplantation in Asian populations: A meta-analysis
Source: PLoS One. 2017 May 4;12(5):e0176827. doi: 10.1371/journal.pone.0176827 (PMC5417567; doi:10.1371/journal.pone.0176827)
Supplement: S1 Table — (DOCX) [file pone.0176827.s001.docx]

| Rsfs. | HR(OS) | LL(OS) | UL(OS) | HR(DFS) | LL(DFS) | UL(DFS) |
| --- | --- | --- | --- | --- | --- | --- |
| Yu et al[12] | 4.28 | 2.58 | 7.09 |  |  |  |
| Satoh et al[9] | 3.48 | 2.14 | 5.67 |  |  |  |
| Shiomi et al[10] | 1.64 | 0.97 | 2.77 |  |  |  |
| Yeh et al[11] | 4.42 | 2.68 | 7.28 | 2.47 | 1.50 | 4.08 |
| Noda et al[7] | 6.15 | 3.91 | 9.69 |  |  |  |
| Shao et al[8] | 3.23 | 2.09 | 5.00 |  |  |  |
| Oba et al[3] | 4.44 | 2.36 | 8.33 |  |  |  |
| Kim et al[13] | 2.60 | 0.78 | 8.61 | 2.04 | 1.04 | 4.00 |
| Wong et al[14] | 0.83 | 0.41 | 1.65 | 1.29 | 0.86 | 1.93 |
| Pang et al[1] | 3.89 | 2.74 | 5.52 |  |  |  |

**S1 Table. HR, LL and UL for studies included in meta-analysis.**

OS: overall survival, DFS: disease-free survival
